# Kalman filter approach to real options with active learning

**DOI:** 10.1007/s10287-022-00423-1

**Published:** 2022-01-27

**Authors:** Sebastian Sund, Lars H. Sendstad, Jacco J. J. Thijssen

**Affiliations:** 1 Department of Industrial Economics and Technology Management, NTNU, 7491 Trondheim, Norway; 2 Management School and Department of Mathematics, University of York, York, YO10 5DD United Kingdom

## Abstract

Technological innovations often create new markets and this gives incentives to learn about their associated profitabilities. However, this decision depends not only on the underlying uncertain profitability, but also on attitudes towards risk. We develop a decision-support tool that accounts for the impact of learning for a potentially risk-averse decision maker. The Kalman filter is applied to derive a time-varying estimate of the process, and the option is valued as dependent on this estimation. We focus on linear stochastic processes with normally distributed noise. Through a numerical example, we find that the marginal benefit of learning decreases rapidly over time, and that the majority of investment times occur early in the option holding period, after the holder has realized the main benefits of learning, and that risk aversion leads to earlier adoption. We find that risk-aversion reduces the value of learning and thus reduces the additional value of waiting and observing noisy signals through time.

## Introduction

Investment decisions, such as the launch of nascent technologies, or whether to enter an emerging market, often entail considerable amounts of uncertainty. For example, the rapid adoption of green hydrogen encompasses both uncertainties regarding technological potential and the future market size (Financial Times 2021), both of which a decision maker can learn more about through actively acquiring information over time. The process of updating a priori expectations when information arrives is known as *learning*. However, the learning process is usually not explicitly considered in standard investment analysis such as net-present value calculations and real options. In this paper we allow for a more realistic learning process, where a decision maker can derive an optimal strategy for irreversible investments, by incorporating noisy signals to discern the true profitability of a potential investment project.

More specifically, we examine the optimal investment behavior of a firm that has the option to invest in a project against a fixed sunk cost, and consider a situation in which the underlying variable of the project value is assumed to be either determined by an unknown profitability constant or the state of an Ornstein-Uhlenbeck (OU) process. The initial state is assumed uncertain, and since the process is not perfectly observable, its value remains uncertain throughout the holding period. The firm may engage in obtaining noisy observations of the process over time in order to estimate its value, and consequently, the project’s value. This noise represents a firm-specific uncertainty, and the firm is assumed to face an incomplete market. We, therefore, resort to maximizing expected utility, assuming a firm with a known constant rate of relative risk aversion and rate of time preference.

We begin our analysis by formulating a continuous-time subjective belief of an OU stochastic process. The resulting estimation procedure is compared with an underlying process that is constant at the mean value of the OU process. We derive the expected net present utility of investing in the project, and proceed to discuss properties of the option value. The optimal investment policy is determined using a simulation-based method. Finally, we present a case study in order to demonstrate the dynamics of the model.

The contribution of our work is threefold. First, we develop a utility-based framework in order to analyse how a decision maker can learn from noisy signals to impact investment decisions under different assumptions regarding the stochastic nature of the problem. Second, we derive analytical results, where possible, for the optimal investment strategy and the associated investment rule. Third, we provide managerial insights for investments with active learning based on a numerical analysis of the model.

The managerial insights are as follows. Firstly, the value of learning decreases in the initial estimate of the state process. Intuitively, if the decision maker has a high initial estimate, its expected value of learning is low, since the investment opportunity is very likely to be profitable anyway. Secondly, value of learning is increasing in the standard deviation of the initial estimate of the state process. Hence, reducing initial parameter uncertainty, through, e.g., market research can significantly reduce the value of learning over time. Thirdly, we find that most of the learning happens early in the option’s life-time. Finally, even though the value of learning increases in the uncertainty over the initial estimate, the means and medians of the investment time distribution shows the opposite result. We think that this is due to a positive relationship between the initial uncertainty and the initial marginal benefit of learning.

The remainder of the paper is organized as follows. We proceed by discussing some related work in Sect. 2, and outline our model in Sect. 3. Finally, a case study is then introduced and discussed in Sect. 4, followed by concluding remarks in Sect. 5.

## Related work

The traditional real options literature address the problem of optimal investment under uncertainty (Dixit and Pindyck 1994; McDonald and Siegel 1985, 1986), and, recently, this literature has been extended by the incorporation of learning. This new methodology considers information acquisition as a conscious activity by the firm, and not a passive consequence of waiting to exercise the option. In contrast to traditional models in which parameters that establish the project value are assumed to be known at the beginning of the option holding period, a learning firm changes its estimates throughout this period, either discretely or continuously, based on received information. This additional benefit of holding the option therefore introduces another quantifiable factor to consider when deciding whether to exercise.

There are several ways of modeling active learning. One widely applied method is by Bayesian updating of a parameter’s prior probability distribution when new observations arrive. Examples of this approach include Singh et al. (2018), who apply Bayesian updating at discrete observations to estimate the drift and diffusion parameters of an OU process, or Blanke and Bosq (2012) who study a similar problem in both discrete and continuous time. Although the majority of papers that model learning in a real options setting do so through Bayesian updating, another equally feasible estimation method is the Kalman filter algorithm, as outlined in, e.g., Øksendal (2013). Instead of sequentially updating the entire probability distribution of the estimated parameter, the Kalman filter algorithm is generally applied to only update the conditional expectation, circumventing some of the analytical difficulties with Bayesian updating. Note that, when the observations are considered to be independent of the observed parameter, and observation noise is considered to be normally distributed, the two approaches result in identical estimates, as demonstrated by Barker et al. (1995) and Soyer (2018).

A familiar result in real options analysis is the non-decreasing relationship between uncertainty and option value. Incorporating learning in a real options framework often reduces this uncertainty, and a natural question is whether learning is valuable in this context. As Martzoukos and Trigeorgis (2001) conclude, the value lost from lower uncertainty is compensated by the value gained from better information. This may also be the case if learning is costly, as supported by the results of Harrison and Sunar (2015) and Bellalah (2001), among others, supporting the notion that incorporating learning adds additional value to the traditional real options framework. In addition, the ideas pursued in this paper are applicable not only to optimal stopping problems, but also models of impulse control, such as, e.g., Dumas (1991) and Peura and Keppo (2005).

Nevertheless, the work on learning in a real options context is relatively scarce. Among the existing literature, Kwon and Lippman (2011), Ryan and Lippman (2003), Kwon et al. (2016) and Thijssen et al. (2004) take a theoretical approach and illustrate how the optimal investment strategy is influenced by different aspects of learning about some aspect of future project profitability when the decision maker is assumed to be able to enter, exit or expand the project, or a combination of the three. Herath and Herath (2008), Kwon (2014) and Dalby et al. (2018) examine how learning with real options can be applied to specific situations in industry. More specifically, Herath and Herath (2008) consider how learning may help in valuing certain types of information security systems, and conclude that the incorporation of learning leads to a reduction of upward bias in estimates, as well as specific implications for security system management. Kwon (2014) models the optimal decision policy of a firm that has the option to invest in order to protect a project against disruption and may continuously learn about the probability of this disruption from trends in the market. His model illustrates the sensitivity of optimal decision to the probability of disruption. Dalby et al. (2018) consider a firm which may invest in a renewable energy project, that is subject to an expected adjustment of the support scheme which is currently present. The firm is assumed to be able to learn about the arrival rate of the adjustment from a continuous information stream. The authors illustrate how the optimal investment threshold varies with the desired learning rate and the corresponding effect on option value, and, notably, how the relative time to optimal investment decreases with learning rate. A key contribution of our paper in the context of the aforementioned papers is that we estimate a stochastic process, and not a parameter that is assumed to be constant in time.

A factor of interest in relation to active learning is the “rate of learning”, or, “learning rate”, represented by Kwon et al. (2016) as a parameter that reflects the magnitude of the difference between the prior and posterior probability distributions when applying Bayesian updating. With a Kalman filter, the equivalent measure would be the magnitude of the difference between the prior and posterior conditional expectations. In both cases, the learning rate may be seen as a function of the volatility of the observation process. This volatility describes the uncertainty of the estimate, and a higher rate of learning is intended to translate to a faster decrease in estimator uncertainty. When learning is considered to be costly, the cost function may be expressed in terms of this learning rate, as demonstrated in Moscarini and Smith (2001), and the investor is consequently faced with selecting the optimal learning rate. We consider a fixed learning rate in this article, but acknowledge the importance of discussing the optimal learning rate when considering practical applications, especially when learning is costly, as discussed in Hagspiel et al. (2019). We assume costless learning to simplify the analysis and to illustrate the dynamics of the optimal strategy more clearly. For further discussion on costly learning within a real options framework, see, e.g., Pertile et al. (2014); Harrison and Sunar (2015); Moscarini and Smith (2001); Bellalah (2001); Bergemann and Välimäki (2008); Keller and Rady (1999) and Thijssen and Bregantini (2017).

We focus our discussion on a certain class of stochastic processes known as OU processes. These processes are mean-reverting, and have been applied to model a wide range of scientific phenomena. Within finance, evidence for mean-reversion is abundant (Wong and Lo 2009), and the OU process has been used to model commodity prices, as in Schwartz (1997) and Lucia and Schwartz (2002), exchange rates, as in Jorion and Sweeney (1996), and interest rates, as in Vasicek (1977). In a real options framework, Ekström et al. (2011) formulate the problem of when to liquidate a position in a pairs trade by modeling a mean-reverting price spread with an OU process. Their model is extended by Leung and Li (2015) with the incorporation of a strategy for optimal entry into the position. In an industrial context, Näsäkkälä and Fleten (2005) analyse a real options problem of investment in a power plant when the spread between the electricity price and cost of gas is assumed to follow the sum of an arithmetic Brownian motion and an OU process, similar to the method applied in Lucia and Schwartz (2002). Overall, if an observable underlying process of a project is an OU, and the decision maker has derived an expression for the expected value of the project, our model may be applied to devise an optimal investment strategy. As an example of current relevance, Gray et al. (2011) demonstrate that the disease transmission coefficient in an epidemiological SIS-model may be expressed by an OU process. If a decision maker formulates a project value in terms of this coefficient, our model may be applied to value the opportunity of investing in it. We introduce a case study that assumes an industry with mean-reverting prices following an OU model. It should be noted, however, that our main concern in this paper is to derive a model of general applicability.

Although the aforementioned literature offers meaningful insights on optimal investment decisions and learning, it is developed under the assumption of risk neutrality, which relies on the assumption that the underlying asset may be spanned or replicated by assets in the market. It has been pointed out by Hugonnier and Morellec (2007) that assumptions of risk neutrality or market completeness may be convenient to characterize investment decisions under uncertainty, they “are not particularly relevant to most real-world applications”. In particular, corporate executives and entrepreneurs typically have to make investment decisions in situations where the cash flows from the project are not spanned by those of existing assets or under other constraints which make them face incomplete markets. In such environments, we can expect their risk aversion to affect firms’ investment decisions. In this paper, the stochastic process underlying the option value consists of a volatility component that changes with time. If the firm were to attempt to create a replicating portfolio, it would have to continuously update the portfolio composition in order to accurately reproduce the dynamics of the process. As noted by Leland (1985) and, more recently, Kolm and Ritter (2019), the presence of transaction costs makes a continuously updated portfolio infinitely costly in theory. In practice, a dynamically replicating portfolio would be updated discretely, which limits total transaction costs, at the expense of a lower replication accuracy. Although there are ways of optimizing this trade-off, as both Leland (1985) and Kolm and Ritter (2019) show, we have decided on a different modelling approach that avoids these difficulties altogether. Similar to Henderson and Hobson (2002), we assume a firm with known, constant relative risk aversion (CRRA), as well as a constant rate of time preference. Following Hugonnier and Morellec (2007), we consider the firm’s net present utility of investing in the project rather than its expected net present value of cash flows as the relevant condition for investment decisions. With this approach, the risk originating from the volatility of the estimated process is incorporated in the valuation of the investment opportunity. It should be noted that the utility function may easily be converted to its risk-neutral equivalent by letting the firm’s risk aversion be equal to zero, in case the estimation is in fact spanned by existing assets. Our model therefore has wider applicability than one that expresses its exercise condition in terms of expected NPV.

Since attitudes towards risk and the ability to learn about market conditions impact the optimal investment policy significantly, we explore their interaction and combined impact in this paper. Our results show how the main benefits from learning occur early in the option lifetime, and that the distribution of exercise times has a positive relationship with both the mean and variance of the distribution of the initial estimate. Furthermore, when the unobserved profitability indicator is assumed to be constant, the decision maker postpones the investment compared to a mean-reverting process. Although increasing uncertainty about the initial profitability estimate increases the incentive to learn, risk-aversion decreases the project’s option value and makes it less attractive to engage in active learning.

## Model

We consider a risk-averse decision maker who has a finitely-lived option to make an irreversible investment in a project for a known and fixed sunk cost $k>0$. The present value of the free cash flows that are thrown off by the project over its life time are modeled as a stochastic *state process* $X=(X_t)_{t\ge 0}$. Uncertainty is modeled on a probability space $(\Omega ,{\mathcal {F}},P)$, which is endowed with a filtration ${\mathbf {F}}=({\mathcal {F}}_t)_{t\ge 0}$. The state process *X* is assumed to take values in ${\mathbb {R}}$ and to be adapted to ${\mathbf {F}}$. The *present value of the free cash flows* (PVFCFs) thrown off by the project are given by a continuous, and increasing function, *F*, of the state variable. The *utility* of the PVFCFs is given by a continuous, increasing, and concave function *U*.

Contrary to standard real options models, we assume that the state process *X* is only noisily observed. This is a realistic assumption when, e.g., the project represents an investment in a new market for which future demand is not observed until investment actually takes place. However, we assume that the decision maker can learn about the state process through these noisy observations, e.g., through ongoing market research into noisy signals. In line with Harrison and Sunar (2015), we assume that information is received by the decision maker frequently enough to be modeled as a continuous process that generates the *information filtration* ${\mathbf {G}}=({\mathcal {G}}_t)_{t\ge 0}$.

The decision maker uses the information filtration to form an estimate of the state of the process at any point in time. We will refer to this estimate as the decision maker’s *estimation process* ${\widehat{X}}=({\widehat{X}}_t)_{t\ge 0}$. At the start of the planning horizon, the decision maker holds an initial estimate ${\widehat{X}}_0$ with non-zero variance. The decision maker’s objective then is to solve the optimal stopping problem

1 $$\begin{aligned} F^*(t,{\widehat{X}}_t) = \sup _{\tau \in [t,T]}{\mathbb {E}}\Big [e^{-\rho (\tau -t)}({\mathbb {E}}[U\circ F(X_\tau )|{\mathcal {G}}_\tau ]-k)\Big |{\mathcal {F}}_t\Big ], \end{aligned}$$

where $\rho >0$ is the decision maker’s time discount rate and the supremum is taken over all ${\mathbf {F}}$-stopping times.

### The estimation process

Since the decision maker continuously receives information about the state process, the value of the estimation process evolves stochastically over time, and consequently so does the decision maker’s expected value of the project. Hence, the option value depends on this expectation which is governed by a stochastic differential equation (SDE) that describes the evolution of the belief process through time.^1^ We begin by introducing the underlying process and the data generating process, and proceed to derive a general SDE for the belief process.

We assume that the state process *X* is the unique strong solution to the stochastic differential equation

2 $$\begin{aligned} dX_t = \mu (X_t, t)dt + \sigma (X_t, t)dB_t, \end{aligned}$$

with given (but unobserved) initial value $X_0$, where $B=(B_t)_{t\ge 0}$ is a standard Brownian motion. The decision maker’s observations are given by the process $H=(H_t)_{t\ge 0}$, with for all $t\ge 0$,

3 $$\begin{aligned} H_t = \beta (X_t, t) + \gamma (X_t, t)W_t, \end{aligned}$$

where $W=(W_t)_{t\ge 0}$ is a standard Brownian motion independent of *B*. By defining the (cumulative) *observation process* $Z=(Z_t)_{t\ge 0}$ as

4 $$\begin{aligned} Z_t = \int _{0}^{t}H_s ds, \end{aligned}$$

it follows that (Øksendal 2013, p. 86) the observation process can be represented in differential form as

5 $$\begin{aligned} dZ_t = \beta (X_t, t)dt + \gamma (X_t, t)dV_t, \end{aligned}$$

where $dV_t=W_t dt$, so that $V=(V_t)_{t\ge 0}$ is a standard Brownian motion independent of *U*.

The *filtering problem* then is: Given observations $Z_s$ satisfying Eq. (5) for $0\le s \le t$, what is the best estimate ${\widehat{X}}_t$ of the state $X_t$ based on these observations? Here, “best” is interpreted in the sense of minimizing mean-squared error. That is, the estimation process ${\widehat{X}}=({\widehat{X}}_t)_{t\ge 0}$ is such that for all $t\ge 0$ it holds that

1. ${\widehat{X}}_t$ is ${\mathcal {G}}_t$-measurable, where ${\mathcal {G}}_t$ is the $\sigma$-algebra generated by observations $(Z_s)_{0\le s\le t}$,
2. $$\mathbb {E}\left[ (X_t - {\widehat{X}}_t) ^2\right] =\inf \left\{ \mathbb {E}\left[ (X_t - Y) ^2\right] : Y\in {\mathcal {K}}\right\} $$, where
  $$\begin{aligned} {\mathcal {K}}_t\equiv \left\{ Y:\Omega \rightarrow {\mathbb {R}}; Y \text { is }{\mathcal {G}}_t\text {-measurable}\right\} . \end{aligned}$$

The best estimate may be expressed as ${\widehat{X}}_t=\mathbb {E}\left[ X_t|\ {\mathcal {G}}_t\right]$ (Øksendal 2013, Theorem 6.1.2). In order to obtain an expression for ${\widehat{X}}_t$ we apply the Kalman filter, which has been applied to a wide range of estimation problems (Grewal 2011). If observations of a certain state are subject to normally distributed measurement inaccuracies, then the Kalman filter allows one to identify the estimator with the smallest mean squared error among candidate estimators. In our model, the noise that arises when measuring $\beta (s, X_s)$ at measurement times $s\in [0,\ t]$ is expressed by the term $\gamma (s, X_s)W_s$ from Eq. (3). Note that, depending on its structure, a measurement of $\beta (s, X_s)$ may be transformed to a measurement of $X_s$.

To keep our analysis as simple as possible, we restrict attention to observations of a linear dynamical system, in which the aforementioned processes take the form

6 $$\begin{aligned} dX_t&= F(t)X_tdt + C(t)dB_t,\quad \text {and} \end{aligned}$$

7 $$\begin{aligned} dZ_t&= G(t)X_tdt + D(t)dV_t, \end{aligned}$$

i.e. $\beta (X_t,t)=G(t)X_t$, $\gamma (X_t, t)=D(t)$, $\mu (X_t, t)=F(t)X_t$, and $\sigma (X_t, t)=C(t)$. As noted by Soyer (2018), a Kalman filter algorithm applied to linear dynamical systems with Gaussian noise results in a Gaussian distribution that is identical to the distribution obtained by application of sequential Bayesian updating. This distribution may consequently be used to obtain expectations of functions of the observed process at a given *t*, either analytically or numerically.

If the linear dynamical system takes the form of Eqs. (6)–(7), (Øksendal 2013, Theorem 6.2.8) shows that the application of a Kalman filter results in a stochastic differential equation for ${\widehat{X}}_t$ of the form

8 $$\begin{aligned} d{\widehat{X}}_t=\left( F(t)-\frac{G^2(t)S(t)}{D^2(t)}\right) {\widehat{X}}_tdt + \frac{G^2(t)S(t)}{D^2(t)}dZ_t, \end{aligned}$$

where ${\widehat{X}}_0=\mathbb {E}[X_0]$, and $S(t)=\mathbb {E}\left[ (X_t-{\widehat{X}}_t)^2\right]$, which satisfies the Ricatti equation

9 $$\begin{aligned} \frac{dS(t)}{dt}=2F(t)S(t)-\frac{G^2(t)}{D^2(t)}S^2(t)+C^2(t). \end{aligned}$$

If functions *F*(*t*), *C*(*t*), *G*(*t*) are solvable analytically, we may derive ${\widehat{X}}_t$ explicitly. In any case, the stochastic differential Eq. (8) is sufficient to derive an option value, and we will therefore focus our attention on this equation. We simplify Eq. (8) by introducing coefficient functions $L_1(t)=F(t)-\frac{G^2(t)S(t)}{D^2(t)}$ and $L_2(t)= \frac{G^2(t)S(t)}{D^2(t)}$, so that

10 $$\begin{aligned} d{\widehat{X}}_t=L_1(t){\widehat{X}}_tdt + L_2(t)dZ_t. \end{aligned}$$

Using (10), we apply our model to two different situations. In the first, the state process *X* is assumed to be constant. In the second, it is assumed to follow a mean-reverting Ornstein-Uhlenbeck (OU) process. For clarity, we denote the coefficient functions of the first application as $L_{1,c}(t)$ and $L_{2,c}(t)$ and those of the second application as $L_{1,o}(t)$ and $L_{2,o}(t)$. In any general discussion we will drop the subscripts.

#### Application 1: A constant process

Consider a filtering problem in which the state process *X* is constant, so that $dX_t = 0$ and $X_t = X_0$, a.s. At time $t=0$ the decision maker has a *prior* estimate ${\widehat{X}}_0={\mathbb {E}}\left[ X_0\right]$ with variance ${\mathbb {V}}\left[ X_0\right] =a^2$. Observations are assumed to be of the form

11 $$\begin{aligned} H_t=X_t+mW_t, \end{aligned}$$

for some $m\in {\mathbb {R}}\setminus \{0\}$, so that

12 $$\begin{aligned} dZ_t=X_tdt+mdV_t. \end{aligned}$$

Following the same steps as for the general case, we obtain the following stochastic differential equation for the best estimate of $X_t$:^2^

13 $$\begin{aligned} d{\widehat{X}}_t = L_{1,c}(t){\widehat{X}}_tdt + L_{2,c}(t)dZ_t. \end{aligned}$$

In this case,

14 $$\begin{aligned} L_{1,c}(t)= -\frac{a^2}{m^2+a^2t},\quad \text {and}\quad L_{2,c}(t)= \frac{a^2}{m^2+a^2t}. \end{aligned}$$

By expanding $dZ_t$ the process ${\widehat{X}}$ can be expressed in terms of the Brownian motion differential, i.e.

15 $$\begin{aligned} d{\widehat{X}}_t = m L_{2,c}(t)dV_t. \end{aligned}$$

#### Application 2: An OU process

Here we consider a filtering problem in which the state process *X* follows an OU process, with $dX_t=-pX_tdt+qdU_t$, for $p> q > 0$. As before, the observer holds an estimate of $X_0$ with a mean of ${\widehat{X}}_0={\mathbb {E}}\left[ X_0\right]$ and variance ${\mathbb {V}}\left[ X_0\right] =a^2$. Following the same steps as in Sect. 3.1.1, we get the following coefficients:^3^

16 $$\begin{aligned} L_{1,o}(t)= & {} -\frac{\left( p+\frac{a^2}{m^2} \right) \sqrt{p^2+\frac{q^2}{m^2}}+\left( p^2+\frac{q^2}{m^2} \right) \tanh {\left( t\sqrt{p^2+\frac{q^2}{m^2}}\right) }}{\sqrt{p^2+\frac{q^2}{m^2}}+\left( \frac{a^2}{m^2}+p \right) \tanh {\left( t\sqrt{p^2+\frac{q^2}{m^2}}\right) }}, \quad \text {and}\qquad \end{aligned}$$

17 $$\begin{aligned} L_{2,o}(t)= & {} \frac{a^2}{m^2}\frac{\sqrt{p^2+\frac{q^2}{m^2}}-\left( p-\frac{q^2}{a^2}\right) \tanh {\left( t\sqrt{p^2+\frac{q^2}{m^2}}\right) }}{\sqrt{p^2+\frac{q^2}{m^2}}+\left( \frac{a^2}{m^2}+p\right) \tanh {\left( t\sqrt{p^2+\frac{q^2}{m^2}}\right) }}. \end{aligned}$$

By expanding $dZ_t$, the process ${\widehat{X}}$ can be expressed in terms of the Brownian motion differential, i.e.

18 $$\begin{aligned} d{\widehat{X}}_t = -p{\widehat{X}}_tdt + m L_{2,o}(t)dV_t. \end{aligned}$$

This shows that the estimation process ${\widehat{X}}$ takes the same form as the state process *X*, albeit with a different diffusion term.

We have so far assumed that the process reverts to zero. However, certain applications require the process to revert to a specific constant $\mu$. Following Hull (2015, Sect. 31.7), without loss of generality, we may shift the process by $\mu$ and analyze ${\widehat{X}}_t + \mu$, while still modeling ${\widehat{X}}_t$ as an OU process reverting to zero. Note that by shifting the OU in such a way, it effectively becomes structurally equivalent to the model in Vasicek (1977), which allows for mean reversion to a nonzero constant. Furthermore, it is worth noting that by allowing $\mu$ to be time dependent such that $\mu =\mu (t)$, we may model observations of processes that are assumed to have a time-dependent long-run mean as the sum of $\mu (t)$ and a non-shifted OU model. This may for example be applicable to situations in which the process is influenced by seasonal effects.

#### Comparisons

As is evident from Eqs. (15) and (18), the function $L_2$ plays a crucial role in how the estimates evolve with time. The component has similar characteristics for both processes. Specifically,

i. $$L_2(0) = \frac{a^2}{m^2}$$,
ii. $$0 \le \underset{t\rightarrow \infty }{\lim } L_2(t) < \infty $$,
iii. $L_2(t) > 0$ and $L'_2(t)<0$ when $t>0$,
iv. $L_2$ is increasing in *a*, and
v. $L_2$ is decreasing in *m*.

This implies that the uncertainty in the initial estimate is equally large for both processes, and decreases strictly towards zero. The negative gradient illustrates how learning affects the estimate, by allowing for greater certainty as time passes. Since $L_2(t)$ is strictly positive as well as strictly decreasing, the gradient must decrease in absolute magnitude with increasing *t*, which may be interpreted as a decreasing marginal benefit of additional observations.

Furthermore, it can be shown that there exists $t'\ge 0$ such that $L_{2,c}(t) \ge L_{2,o}(t)$ if, and only if, $0 \le t \le t'$. The intercept $t'$ exists due a non-zero limiting value of $L_{2,o}$. Specifically,

19 $$\begin{aligned} \underset{t\rightarrow \infty }{\lim } L_{2,c}(t)&= 0,\quad \text {and} \end{aligned}$$

20 $$\begin{aligned} \underset{t\rightarrow \infty }{\lim } L_{2,o}(t)&= \frac{a^2}{m^2}\frac{\sqrt{p^2+\frac{q^2}{m^2}}-\left( p-\frac{q^2}{a^2}\right) }{\sqrt{p^2+\frac{q^2}{m^2}}+\left( \frac{a^2}{m^2}+p\right) } > 0. \end{aligned}$$

The latter result follows from the observation that the uncertainty in the estimation process can never be completely eliminated if the state process is stochastic. Note that if $q=0$, then $L_{2,o}(t)$ does in fact converge to zero. However, we only consider $p> q > 0$, and the uncertainty in the estimation process is, therefore, initially higher for the constant process than for the OU process. This is reversed at time $t'$, after which the uncertainty in the estimation process is higher for the OU process than for the constant process. Note that whether $t' < T$ depends on parameters, so that it is possible that uncertainty in estimating the constant process is higher than uncertainty in estimating the OU process over the project’s entire life-time .

Considering the fact that *m* can be thought of as representing the volatility of individual observations, the fact that $L_2$ decreases in *m* may be surprising. However, when the estimation process is expressed in terms of the Brownian motion differential, the volatility is given by $mL_2(t)$, and it becomes apparent that the estimation process has a volatility that is increasing in *m*.

### The option to invest

Recall that the decision maker’s objective is to solve the optimal stopping problem 1,

$$\begin{aligned} F^*(t,{\widehat{X}}_t) = \sup _{\tau \in [t,T]}{\mathbb {E}}\Big [e^{-\rho \tau }({\mathbb {E}}[U\circ F(X_\tau )|{\mathcal {G}}_\tau ]-k)\Big |{\mathcal {F}}_t\Big ], \end{aligned}$$

where $U:{\mathbb {R}}\rightarrow {\mathbb {R}}$ is an increasing and concave Bernoulli utility function, $\rho >0$ is the decision maker’s time discount rate, $k>0$ is the sunk cost of investment, and $T<\infty$ is the option’s life time. The estimation process represents, at any time $t\in [0,T]$, the decision maker’s best estimate ${\widehat{X}}_t$ (in terms of minimized mean-squared error) of the present value of the project’s future stream of free cash flows, $X_t$.

Since the value of the option depends on the estimation process ${\widehat{X}}$ and if the utility function *U* is differentiable, then it follows from the general theory of optimal stopping (Krylov 1980) that the optimal investment policy will be characterized by a continuous *exercise boundary* $t\mapsto {\widehat{X}}^*_t$, in the sense that the optimal time to invest is the first exit time from the *continuation set*

21 $$\begin{aligned} {\mathcal {C}}= \left\{ \left( t,{\widehat{X}}_t\right) \in [0,T]\times {\mathbb {R}}:{\widehat{X}}_t<{\widehat{X}}^*_t\right\} . \end{aligned}$$

Note that computing the option’s *exercise value*,

22 $$\begin{aligned} \Psi \left( t,{\widehat{X}}_t\right) := {\mathbb {E}}[U\circ F(X_\tau )|{\mathcal {G}}_t]-k, \end{aligned}$$

requires knowledge about the distribution of $X_t$. We use a simulation procedure to estimate this distribution, based on realizations of the estimation process.^4^ For further reference we define a belief process of the form

23 $$\begin{aligned} d{\widehat{X}}_t=L_1(t){\widehat{X}}_tdt+L_2(t)dZ_t, \end{aligned}$$

the *characteristic operator* on $C^2$ as

24 $$\begin{aligned} {\mathcal {A}}f(t,x) = \frac{\partial f(t,{\widehat{X}}_t)}{\partial t} +[L_1(t)+L_2(t)] {\widehat{X}}_t \frac{\partial f(t,{\widehat{X}}_t)}{\partial x}+\frac{1}{2}m^2L_2^2(t) \frac{\partial ^2 f(t,{\widehat{X}}_t)}{\partial x^2}.\nonumber \\ \end{aligned}$$

From the general theory of optimal stopping (Peskir and Shiryaev 2006) it follows that the value function *V* should be $C^1$, $C^2$ a.e., and solve the *variational inequalities*

25 $$\begin{aligned} \max \{\Psi ({\widehat{X}}_t,t)-F^*({\widehat{X}}_t,t),{\mathcal {A}}F^*({\widehat{X}}_t,t)-\rho F^*({\widehat{X}}_t,t)\} =0, \quad \text {for all }(t,{\widehat{X}}_t)\in [0,T]\times {\mathbb {R}}.\nonumber \\ \end{aligned}$$

The corresponding continuation region is given by

$$\begin{aligned} {\mathcal {C}}= \{(t,{\widehat{X}}_t)\in [0,T]\times {\mathbb {R}}: F^*({\widehat{X}}_t,t)>\Psi ({\widehat{X}}_t,t)\}. \end{aligned}$$

Note that (25) implies that on ${\mathcal {C}}$ it should hold that

26 $$\begin{aligned} {\mathcal {A}}F^*(t,{\widehat{X}}_t)-\rho F^*(t,{\widehat{X}}_t) = 0. \end{aligned}$$

This is generally referred to as the *Bellman equation*.^5^

**Fig. 1:**
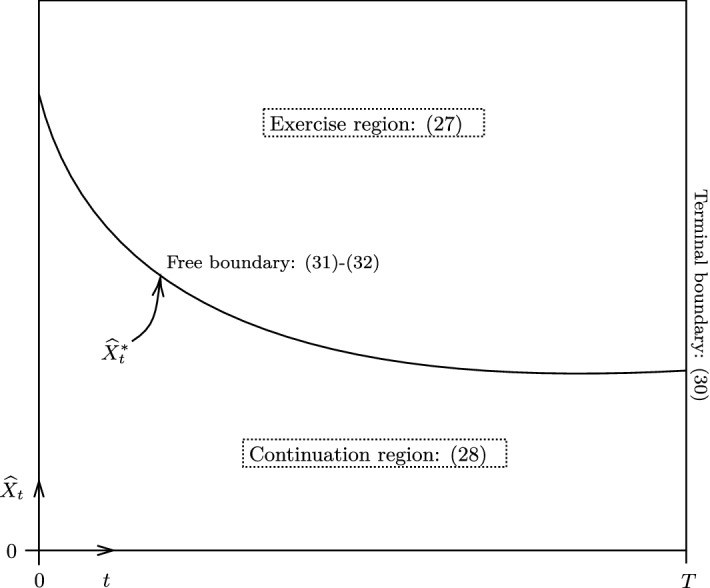
Domain of the option value function $f({\widehat{X}}_t, t)$

Figure 1 summarizes the boundary value problem. Since the free boundary $t\mapsto {\widehat{X}}_t^*$ is initially unknown, it must be identified together with the option value function $F^*(t,{\widehat{X}}_t)$ in the solution procedure. The variation inequalities (25) apply across the entire domain of *V*. Hence, we seek to identify a free boundary $t\mapsto {\widehat{X}}_t^*$ that satisfies

27 $$\begin{aligned} \Psi (t,{\widehat{X}}_t)-F^*(t,{\widehat{X}}_t)&=0 \quad \text {when }{\widehat{X}}_t\ge {\widehat{X}}_t^*, \text { and} \end{aligned}$$

28 $$\begin{aligned} {\mathcal {A}}F^*(t,{\widehat{X}}_t)-\rho F^*(t,{\widehat{X}}_t)&=0\quad \text {when } {\widehat{X}}_t<{\widehat{X}}_t^*, \end{aligned}$$

for all $t\in [0,\ T]$. These constraints, together with the boundary Conditions (29)–(32) below, are sufficient to identify the free boundary and the option value function.

29 $$\begin{aligned}&\lim _{{\widehat{X}}_t\rightarrow -\infty } F^*(t,{\widehat{X}}_t) = 0, \end{aligned}$$

30 $$\begin{aligned}&F^*(T,{\widehat{X}}_{T}) = \Psi (T,{\widehat{X}}_{T}) , \end{aligned}$$

31 $$\begin{aligned}&F^*(t,{\widehat{X}}_t^*) = \Psi (t,{\widehat{X}}_t^*) \quad \text {and} \end{aligned}$$

32 $$\begin{aligned}&\frac{\partial F^*(t,{\widehat{X}}_t^*)}{\partial {\widehat{X}}_t} = \frac{\partial \Psi (t,{\widehat{X}}_t^*)}{\partial {\widehat{X}}_t}. \end{aligned}$$

Condition (29) is a standard “no bubble” condition that states that the option is worthless if the estimation process becomes unboundedly negative. The estimation process giving, at any time *t*, an unbiased estimator with a finite variance, the limit implies that the observed process also approaches negative infinity in expectation. Due to the strictly increasing nature of the exercise value, the option value consequently approaches its minimum value, zero. Condition (30) states that the decision maker makes a now-or-never investment decision at the expiration time *T* of the option. See Dixit and Pindyck (1994, Chapter 3) for intuitive motivations why the *value-matching* Condition (31) and *smooth-pasting* Condition (32) are necessary to ensure an optimal free boundary.

## Results

This section presents an illustration of the model described in Sects. 3. In particular, model the PV of FCFs as

33 $$\begin{aligned} F\left( x\right) = Q\left( x + \mu \right) , \end{aligned}$$

where $Q>0$ is the quantity sold, *x* is the per-unit free cash flow, and $\mu$ is the deterministic long run mean of the price process. We assume that the decision maker’s preferences exhibit constant relative risk aversion (CRRA) and are represented by the utility function

34 $$\begin{aligned} U(w) = {\left\{ \begin{array}{ll} \frac{w^{1-\gamma }}{1-\gamma }\quad \text {when } 0\le \gamma <1, \text { and}\\ \ln (w)\quad \text {when } \gamma = 1, \end{array}\right. } \end{aligned}$$

where $\gamma$ is the Arrow-Pratt coefficient of relative risk aversion. Note that *U* is strictly concave in *w* for any $\gamma >0$, and linear for the case $\gamma =0$ where the decision maker is risk neutral.

The free-boundary problem described there is solved by using the simulation-based approach introduced in Longstaff and Schwartz (2001), in which various American options are valued by first simulating a series of trials of the underlying stochastic process, then obtaining stopping rules for each trial, and finally averaging over the discounted exercise values. We start by calculating the exercise values at the terminal boundary *T*, and thereafter stepping backwards in time, using previously computed exercise values as regressands, and current estimation process values as regressors, in order to obtain least-squares coefficients of a conditional expectation function for the continuation value of the option at the given point in time, which is then compared to the value of immediate exercise. As a useful comparison, this method may be viewed as analogous to the decision problem presented by the Bellman equation in Sect. 3, in which the expected continuation value is compared to the exercise value in continuous time. As noted by Longstaff and Schwartz (2001), the functions used in the regression need to form an orthonormal basis. By increasing the number of these orthonormal basis functions, the accuracy of the procedure can be improved.

If we denote the magnitude and number of time-steps by $\Delta t$ and $N = \lfloor T/\Delta t\rfloor$, respectively, then the regression equation at time-step $j\in \{1,\ \dots ,\ N-1\}$ can be written as

35 $$\begin{aligned} Y(\omega _i, j\Delta t) = \sum _{k=0}^{B} a_{n,j} {\mathcal {L}}_n({\widehat{X}}_j), \end{aligned}$$

where $\omega _i$ indicates the sample path of the *i*-th trial, *Y* indicates the regressand, *B* denotes the number of basis functions, $\{a_{n,j}\}$ the regression coefficients and ${\mathcal {L}}_n$ the *n*th order basis function.^6^

More specifically, we simulate *S* sample paths of the estimation process by the Euler method, discretizing the expanded SDE (23), such that

36 $$\begin{aligned} {\widehat{X}}_j = {\widehat{X}}_{j - 1} + [L_1(t - \Delta t) + L_2(t - \Delta t)]{\widehat{X}}_{j - 1} \Delta t + mL_2(t - \Delta t)\sqrt{\Delta t}\ \zeta _j,\nonumber \\ \end{aligned}$$

where $j=\{1,\ \dots ,\ N\}$ and $\zeta _j \sim {\mathcal {N}}(0, 1)$. All sample paths begin at the initial belief ${\widehat{X}}_0$. In order to reduce the computational cost of the algorithm, we apply antithetic variates when sampling the standard normal distribution. For each trial *i* with a given vector of realized standard normal variables $\{\zeta _{i,j}\}_j$ we design a second trial $i'$ with antithetical realizations $\{-\zeta _{i',j}\}_j$. This results in a negative covariance between path values of trials *i* and $i'$ at any given *j*, which, when applied to all trials, reduces the variance across all path values at *j*, resulting in a lower required number of trials for a desired level of accuracy.

**Table 1:** Base parameter values under risk neutrality and parameters under risk aversion in parenthesis when different. (NOK stands for Norwegian Kroner.)

Parameter	Value	Unit	Description
*T*	5	Years	Option lifetime
*k*	5000	MNOK	Investment cost
*Q*	100	MQ	Proportionality of project value function
$\gamma$	0 (0.1)	–	Relative risk aversion
$\rho$	0.05	–	Rate of time preference (discount rate)
${\widehat{X}}_0$	40	NOK/Q	Initial belief
*a*	60	NOK/Q	Volatility of initial belief
*m*	20	NOK/Q	Volatility of observations
*p*	0.05	Years^-1^	Mean reversion of observed process
*q*	0.02	NOK/Q	Volatility of observed process
$\mu$	40	NOK/Q	Mean of observed process
$\bar{\epsilon }$	0.1%	–	Error tolerance
$\Delta t$	0.05	Years	Magnitude of time discretizations
$\Delta x$	0.2	NOK/Q	Magnitude of integral eval. discretizations
*S*	500,000	–	Number of trials
*B*	5 (6)	–	Number of Laguerre basis functions
$\eta$	16 (10)	–	Order of polynomial smoothing function

We use the parameter values in Table 1 as a base case, and proceed to look at how different properties change with variations of specific parameters.^7^ We separate our discussion into a risk-neutral and risk-averse case, respectively. The risk-neutral case removes a layer of complexity by allowing for a simpler exercise value function, and is included to better illustrate the properties of the model. The risk-averse case is then discussed in terms of deviations from the risk-neutral case. We focus our attention on the free boundary, the value of learning, and expected exercise times.

### Under risk neutrality

Risk neutrality, i.e. $\gamma = 0$, simplifies the structure of the exercise value function and clarifies certain characteristics of the option. Specifically,

37 $$\begin{aligned} \Psi (t,{\widehat{X}}_t) = Q\left( {\widehat{X}}_t + \mu \right) -k, \end{aligned}$$

which is simply the expected net present value of investing.

In order to estimate the option value, we begin with selecting the number of Laguerre basis functions *B* to regress on by analyzing the relative changes in option values for increasing *B*. Selecting $B = 5$ ensures that the simulated option value changes by less than $\bar{\epsilon } = 0.1\%$ when $B = 6$, which we have deemed a high enough accuracy for the purpose of this case study (see Appendix 7). It should be noted that computational limitations such as the number of trials *S* and the magnitude of discrete time steps $\Delta t$ naturally also affect the accuracy of the results. We have consequently selected *M* iteratively, such that for $\Delta t = 0.05$ and $\Delta x = 0.2$, $S = 500{,}000$ trials also gives an option value that changes by less than $\bar{\epsilon } = 0.1\%$ when using $S + 1$ trials.

**Fig. 2:**
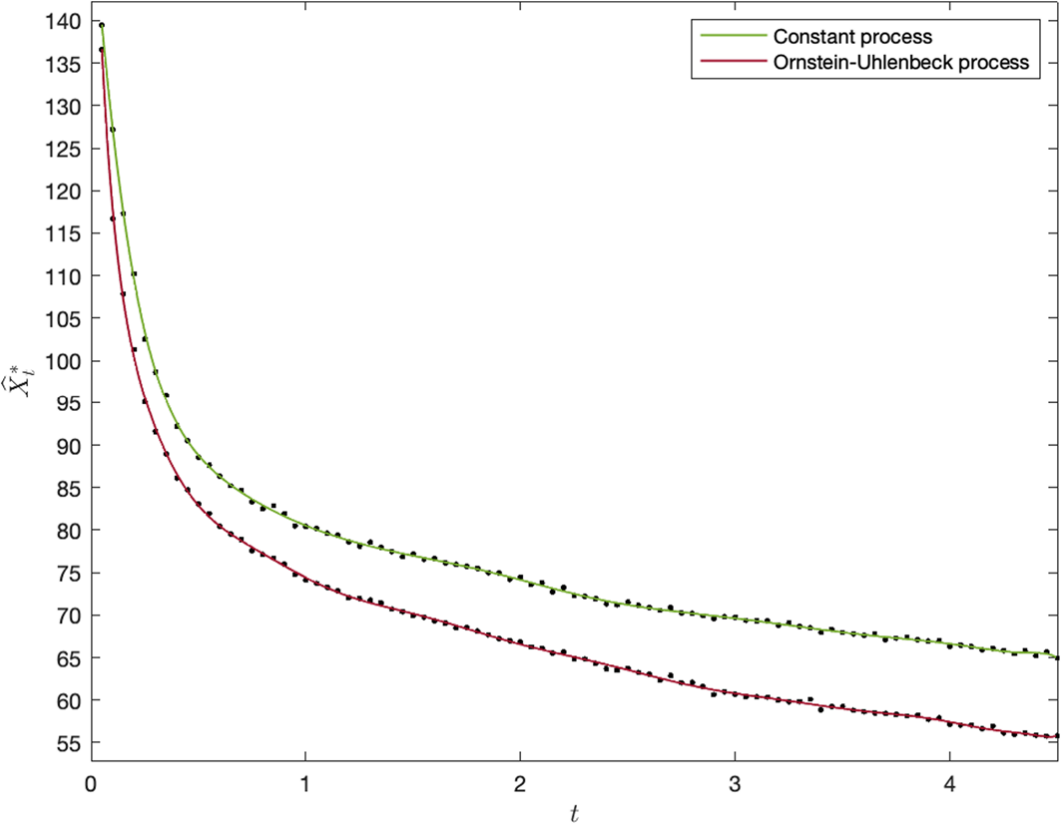
Exercise thresholds (left panel) and the value of learning against ${\widehat{X}}_0 - \mu$ (right panel)

Figure 2 illustrates the free boundary for both the shifted OU process and the constant process, with initial values ${\widehat{X}}_0$ equal to the long-term average $\mu$ of the processes. The discrete points have been smoothed by a polynomial function.^8^ As both cases illustrates, the lower optimal investment threshold indicates a diminishing value of learning. This is because as the decision maker obtains better estimates of the underlying process as time goes by, as is also shown analytically by the decreasing marginal benefit of new observations in Sect. 3.1.3.^9^

Interestingly, the shifted OU process has a lower free boundary than the constant process. This may seem counter intuitive since one the constant process seems less risky, which, in turn, would suggest an earlier adoption due to the reduced value of waiting. However, the OU process reverts back to a known value, and, therefore, will not stray too far away from its true value over time, whereas the constant process has no such convergence. This has crucial implications for a decision maker considering to enter a market. For example, some markets, such as shipping and commodity markets, can have persistent imbalances between supply and demand creating so called super cycles. Here an OU process will be more appropriate, and the decision maker can learn the true state of the market relatively quickly which results in an earlier investment. In contrast, markets where supply gluts are likely to be satisfied promptly due to new entrants, a constant model is a better assumption. This can be applicable for decision makers considering a nascent-online market where barriers to entry are weak.

**Fig. 3:**
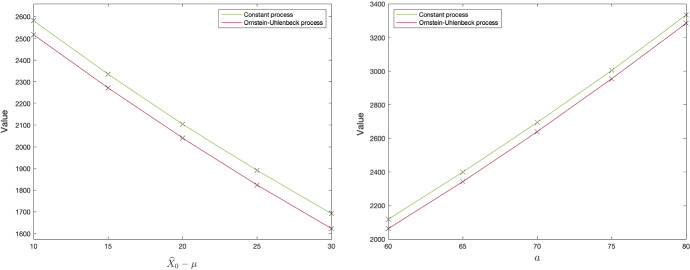
value of learning for different values of *a*

A quantity of managerial interest is the value of learning as opposed to investing immediately. We can measure this as the difference between the option value $F^*(0,{\widehat{X}}_0)$ and the exercise value $\Psi (0,{\widehat{X}}_0)$ at the beginning of the starting time. The left panel of Fig. 3 shows the expected value of learning for an increasing difference between the estimated initial values ${\widehat{X}}_0$ and $\mu$, with the latter held constant. The negative relationship implies that the value of learning decreases with a higher initial estimate of the underlying process. Note that, the option value alone has an increasing relationship with ${\widehat{X}}_0$, but as the figure shows, this trend is offset by an increase in the value of immediate exercise. Intuitively, if the firm has a high initial estimate, its expected value of learning is low, since the investment opportunity seems likely to be profitable in many circumstances. The vertical gap between the constant and OU case is thought to arise because of the difference in magnitude between the volatilities of the processes, as discussed in Sect. 3.1.3. The constant process has a higher volatility than the OU process throughout the holding period, and the value of learning is consequently higher.

The right panel of Fig. 3 shows the expected value of learning against the standard deviation of the initial estimate, *a*. This parameter measures the uncertainty around the initial estimate. As the graph illustrates, greater initial uncertainty leads to a greater option value. Hence, reducing initial parameter uncertainty, through for example market research, can significantly reduce the value of learning over time and, thus, lead to earlier investment. In fact, this property can be established analytically. The proof can be found in Appendix 6.

#### Proposition 1

Suppose that ${\tilde{a}}>a$ and denote the optimal value functions under *a* and ${\tilde{a}}$ by $F^*$ and ${\tilde{F}}^*$, respectively. Then ${\tilde{F}}^*\ge F^*$.

**Fig. 4:**
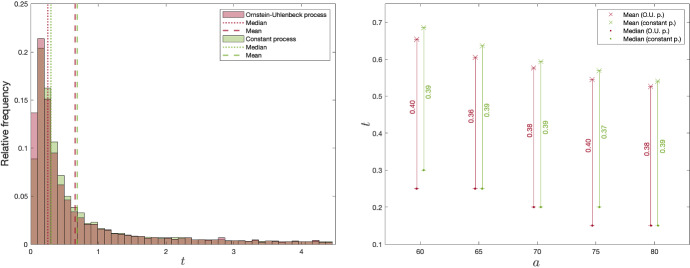
Distribution of exercise times in the risk-neutral case, conditional on exercise before *T*, with corresponding medians and means (left panel). Means and medians of exercise times, conditional on exercise before *T*, against *a* (right panel)

The left panel of Fig. 4 shows the distribution of exercise times conditioned on the trial having been exercised before option termination. The histogram shows a clear trend towards early exercise, with medians 0.25 and 0.30. This result is more intuitive when viewed together with the discussion in Sect. 3.1.3, which examines the properties of the volatilities of the observation processes. Due to the structure of the volatilities, we can conclude that most of the learning happens early in the holding period. The remaining benefit of waiting, as opposed to exercising, is therefore generally at its highest early in the holding period. Notice that the histograms do not start out at their maximum values, but rather increase after some time has passed since $t=0$. This illustrates how the investor is generally too uncertain at the outset of the holding period to forego the option to wait and learn for the base parameter values. With a high initial marginal benefit of learning, however, the estimations improve quickly, and the remaining benefit of waiting decreases, which is thought to explain the skewness. With such a heavy skewness there is a significant distance between the means and medians. This can be an important observation to an institution that makes policy decisions based on market trends, for example, to not only consider the mean time to investment.

Note that, with base parameter values, approximately $40.7\%$ and $44.0\%$ of the trials were exercised for the constant process and OU process, respectively. Intuitively, the decision-maker quickly obtains the necessary parameter certainty and then decides whether to invest or to postpone.

In the right panel of Fig. 4, we show the effects of increasing uncertainty in the initial estimate on the means and medians of the distribution of investment times. Interestingly, even though the value of learning increases with higher *a* (as discussed in relation to Fig. 3), the means and medians show the opposite trend. This shows that there is increasing skewness to the left with higher *a*. This is potentially due to the the positive relationship between the initial uncertainty and the initial marginal benefit of learning. With a higher initial uncertainty, the firm is more likely to change its estimate and, thus, has a steeper gradient in the volatility of the estimation process. We therefore expect the remaining value of learning to decrease faster over time with higher *a*, and consequently result in earlier exercise times.

### Under risk aversion

Next, we consider a risk-averse decision maker with the same investment opportunity as previously analyzed. However, here the number of necessary basis functions to approximate the value function is 6 to satisfy $\bar{\epsilon }$, as illustrated in Appendix 7.^10^

**Fig. 5:**
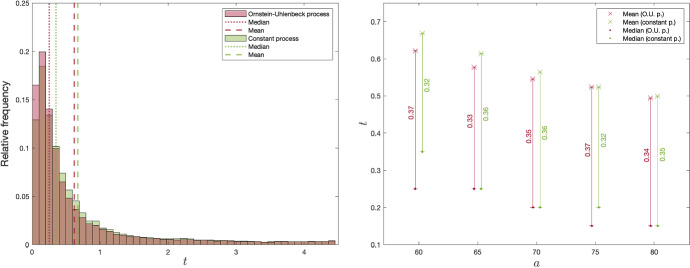
Distribution of exercise times in the risk-averse case, conditional on exercise before *T*, with corresponding medians and means (left panel). Means and medians of exercise times, conditional on exercise before *T*, against *a* (right panel)

To facilitate comparisons, Fig. 5 depicts the same features of the distribution of exercise times as Fig. 4 for the risk-neutral case. Again, we observe skewness in the distribution, slightly lower means and similar medians. This shows that the distribution under risk-aversion is somewhat more skewed under risk-aversion than under risk-neutrality. The intuition for this result is that a more risk-averse decision maker is more willing to “resolve” the uncertainty by investing. This effect is particularly visible for higher values of uncertainty around the initial estimate. This makes intuitive sense: the more uncertainty there is around the estimate of the project’s value, the more eager a risk-averse investor is to remove the uncertainty.

In order to investigate these issues further we simulate 50 scenarios where we choose *q*, *p*, $\gamma$, and *a* uniformly and independently from the intervals [0.01, 0.1], [*q*, 1.25], [0, 0.5], and [60, 180], respectively. The other parameter values are chosen as in Table 1. We then simulate the invest-time distribution and regress the ratio of the third centralized moment and the cubed standard deviation of this distribution on the parameters *q*, *p*, $\gamma$, *a*, and a constant. The $R^2$ of this regression is 0.8339 and the coefficient belonging to *a* is 0.0275 with a *t*-value of 31.48, from which we conclude that the skewness of the investment-time distribution is, indeed, increasing in the volatility of the initial estimate, *a*. Also note that the coefficient of $\gamma$ is 2.3075 with a *t*-value of 14.17, from which we conclude that the skewness of the invest-time distribution is increasing in risk aversion.

**Fig. 6:**
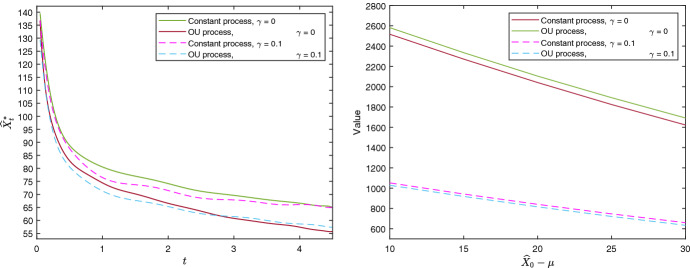
Exercise thresholds (left panel), and values of waiting to learn against ${\widehat{X}}_0 - \mu$ (right panel)

Figure 6 illustrates the investment thresholds under risk neutrality and risk aversion in the left panel, while the right panel illustrates the value of learning under risk aversion, defined as $F^*\left( 0,{\widehat{X}}_0\right) -\Psi \left( 0,{\widehat{X}}_0\right)$. According to the left panel, risk aversion induces earlier investment compared to the risk neutral case. This is in contrast to traditional results, such as Hugonnier and Morellec (2007), who show the opposite result under risk aversion. However, they consider price uncertainty and not active learning. Thus, we conclude that the impact of risk aversion is ambiguous depending on the source of underlying uncertainty.^11^

The right panel of 6 illustrates that the initial value of learning is in fact less valuable under risk aversion than under risk neutrality in our case study. Intuitively, greater risk-aversion decreases the value of the option to learn by more than the current now-or-never investment opportunity, and, thus, reduce the value of learning. Hence, risk aversion may induce earlier investments since the decision maker is less willing to learn and update their prior belief.

### Robustness

To show (numerically) that our results are robust against parameter values, we consider some further numerical experiments in Appendix 8. These show that our qualitative conclusions are not due to the specific parameter values of our base case.

## Conclusion

This article extends real options literature by introducing the ability to learn about a stochastic process underlying the project value with a risk averse decision maker. We analyse how risk aversion interacts with the incentive to learn to impact decisions under both a constant and mean reverting noisy profitability signal. The analysis is motivated by three main features of the modern economic environment: (i) a persistent need to innovate and enter new markets to sustain profitability; (ii) market incompleteness and attitudes towards risk; (iii) parameter uncertainty of investment decisions in emerging technologies. We incorporate these features into a utility-based real-options framework, where a decision maker decides when to invest. Specifically, we assume that the decision maker can learn about the underlying profitability, and, consequently, make a more informed investment decision by actively updating her belief.

We demonstrate how the exercise threshold changes with the marginal value of learning, and how the marginal value of learning is consistently higher for a constant process than an OU process. Numerical results indicate that the value of learning has a negative relationship with the initial process estimate, and a positive relationship with the uncertainty of its distribution. We also show how the value of learning seemingly decreases when the investor is assumed to be risk averse as opposed to risk neutral. These results are robust against changes in the specific parameter values of our base-case scenario.

Furthermore, we identify three potential areas of further research. The first is to relax the continuity assumption of observations, allowing the decision maker to incorporate observations at discrete times. This allows the model to be applied to settings in which observations happen less frequently. The second is to let the project value depend on multiple stochastic processes, all subject to noisy observations. This opens a range of possible extensions such as, for example, a stochastic investment cost that is not directly observable. A third suggestion is to apply the model to a logarithmic transform of a geometric Brownian motion. This allows for a greater range of applications, as geometric Brownian motion is applied extensively within finance, notably to model the behaviour of stock prices. Such a model would, however, not be stationary and would, therefore, require some careful re-calibration of the functions $L_1$ and $L_2$ (cf. Eqs. 14–17). In fact, in such models it is possible that $L_2'>0$, which implies that the observed system diverges from the “true” system.^12^

A final interesting future application for our approach is the recent Covid-19 outbreak. For example, the ratio between infected and fatalities might be expected to be constant in an unvaccinated population, yet unknown. Hence, a decision maker can learn more about the true ratio through several studies as done by (O’Driscoll et al. 2021), and, consequently, adopt appropriate costly measures to limit the impact of Covid-19. Furthermore, the reproduction number of infectious diseases, i.e. how many one infected person will infect, can be considered mean reverting (Gray et al. 2011; Wang et al. 2018). Hence, our framework lends itself well to several other novel applications.

## Figures and Tables

**Fig. 7:**
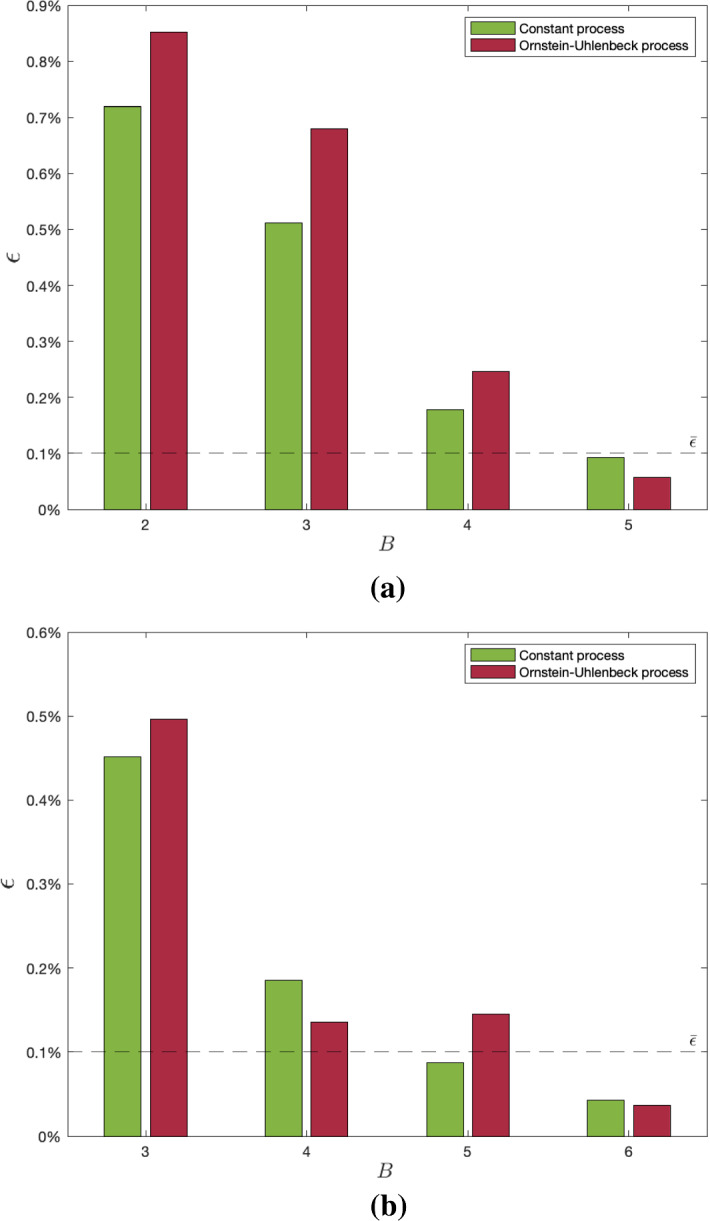
Relative errors of option values $\epsilon$ against the number of basis functions *B* under **a** risk neutrality, and **b** risk aversion

**Fig. 8:**
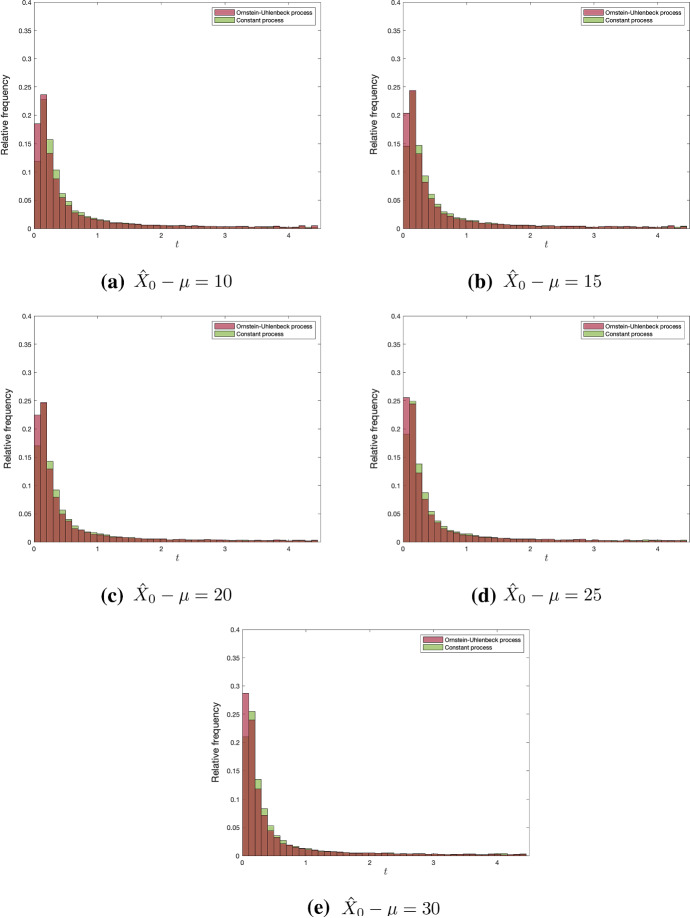
Exercise time distribution for different values of $\hat{X_0}-\mu$ in the risk-neutral case

**Fig. 9:**
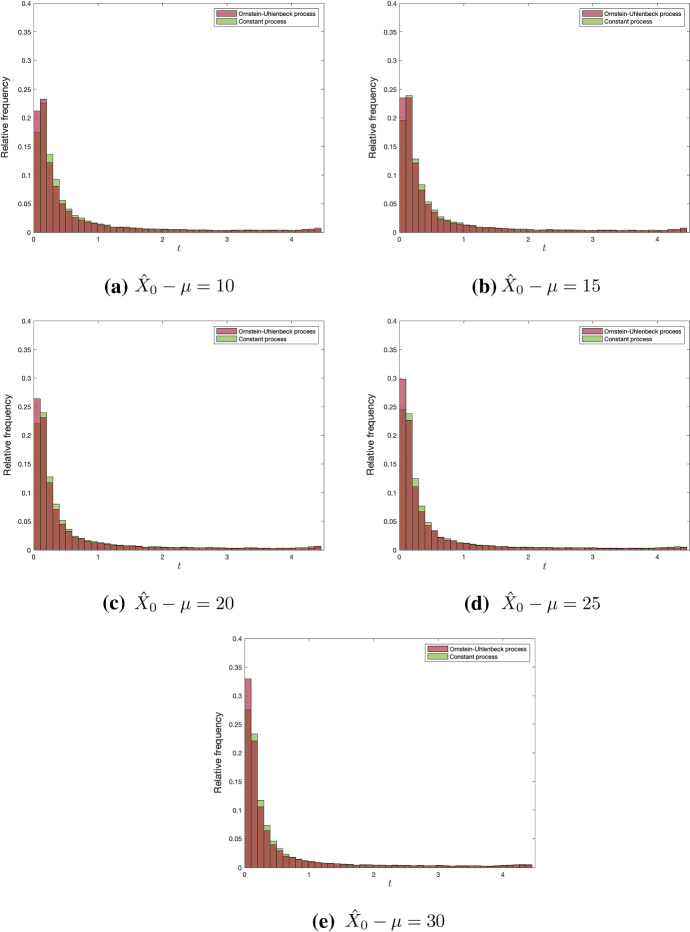
Exercise time distribution for different values of $\hat{X_0}-\mu$ in the risk-averse case

**Fig. 10:**
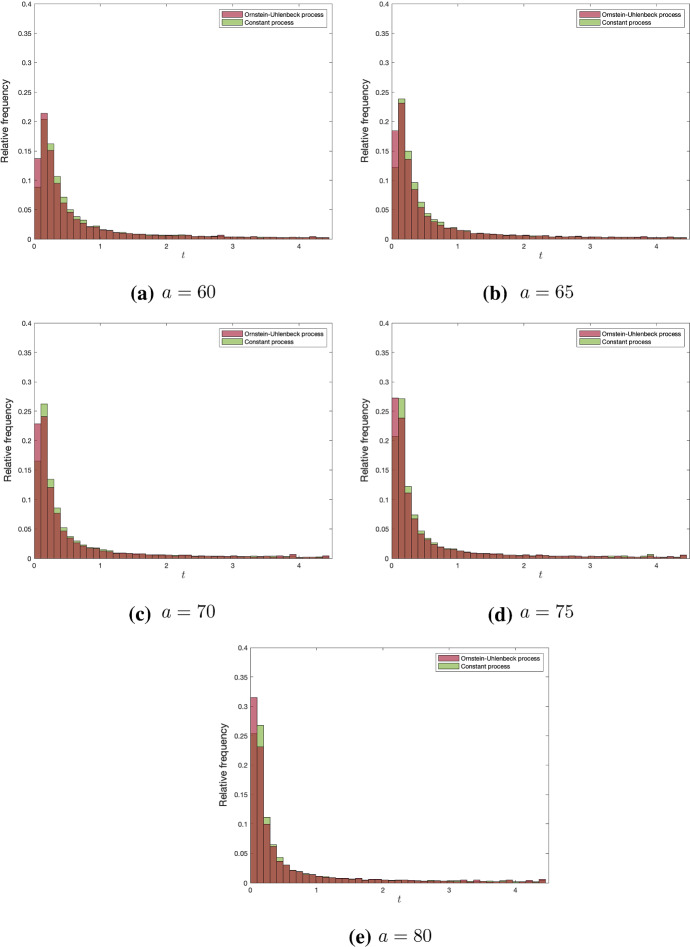
Exercise time distribution for different values of *a* in the risk-neutral case

**Fig. 11:**
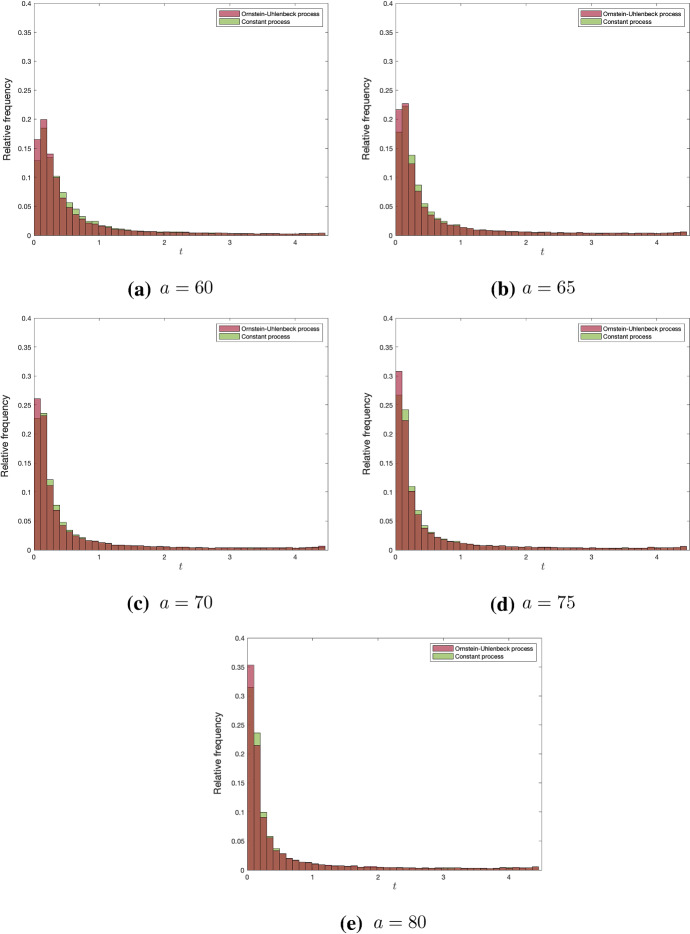
Exercise time distribution for different values of *a* in the risk-averse case

## Appendix

### A Deriving the belief process for a constant parameter

We first solve the ordinary differential equation (ODE) of *S*(*t*) given in Eq. (9) with substitutions $F(t)=C(t)=0$, $G(t)=1$ and $D(t)=m$, which simplifies to

38 $$\begin{aligned} dS(t)=-\frac{1}{m^2}S^2(t)dt,\quad \text {where } S(0)=a^2, \end{aligned}$$

resulting in

39 $$\begin{aligned} S(t)=\frac{m^2a^2}{m^2+a^2t}. \end{aligned}$$

Building on Eq. (8), we obtain

40 $$\begin{aligned} L_{1,c}(t)&= -\frac{a^2}{m^2+a^2t}\quad \text {and} \end{aligned}$$

41 $$\begin{aligned} L_{2,c}(t)&= \frac{a^2}{m^2+a^2t}. \end{aligned}$$

### B Deriving the belief process for a parameter following an OU process

We first solve differential Eq. (9) with substitutions $F(t)=-p$, $C(t)=q$, $G(t)=1$ and $D(t)=m$. This results in

42 $$\begin{aligned} \frac{dS(t)}{dt}=-2pS(t)-\frac{1}{m^2}S^2(t)+q^2,\quad \text {where } S(0)=a^2, \end{aligned}$$

which gives

43 $$\begin{aligned} S(t)=a^2\frac{\sqrt{p^2+\frac{q^2}{m^2}}-\left( p-\frac{q^2}{a^2}\right) \tanh {\left( t\sqrt{p^2+\frac{q^2}{m^2}}\right) }}{\sqrt{p^2+\frac{q^2}{m^2}}+\left( \frac{a^2}{m^2}+p\right) \tanh {\left( t\sqrt{p^2+\frac{q^2}{m^2}}\right) }}. \end{aligned}$$

Building on Eq. (8), we obtain

44 $$\begin{aligned} L_{1,o}(t)&= -\frac{\left( p+\frac{a^2}{m^2} \right) \sqrt{p^2+\frac{q^2}{m^2}}+\left( p^2+\frac{q^2}{m^2} \right) \tanh {\left( t\sqrt{p^2+\frac{q^2}{m^2}}\right) }}{\sqrt{p^2+\frac{q^2}{m^2}}+\left( \frac{a^2}{m^2}-p \right) \tanh {\left( t\sqrt{p^2+\frac{q^2}{m^2}}\right) }}\quad \text {and} \end{aligned}$$

45 $$\begin{aligned} L_{2,o}(t)&= \frac{a^2}{m^2}\frac{\sqrt{p^2+\frac{q^2}{m^2}}-\left( p-\frac{q^2}{a^2}\right) \tanh {\left( t\sqrt{p^2+\frac{q^2}{m^2}}\right) }}{\sqrt{p^2+\frac{q^2}{m^2}}+\left( \frac{a^2}{m^2}+p\right) \tanh {\left( t\sqrt{p^2+\frac{q^2}{m^2}}\right) }}. \end{aligned}$$

### C The expected value function

As noted in Sect. 3, when the Kalman filter is applied to a system of linear state and observation processes with Gaussian sources of uncertainty, the best estimators have a Gaussian distribution that is equivalent to the distribution of the observed process conditional on historic observations. Hence, we seek the expectation and variance of this distribution at time *t*, given previous observations, for each of the model applications.

The expected value of the estimation process is already established as ${\mathbb {E}}[X_t|{\mathcal {G}}_t] = {\widehat{X}}_t$. For both observation processes, regardless of the initial ${\widehat{X}}_0$, the variance equals the integrated volatility of the process since observations began. This is equivalent to the variance of the observed process conditioned on previous observations. For the constant process, we first integrate (15) to obtain

46 $$\begin{aligned} {\widehat{X}}_t = \int _0^{t}mL_{2,c}(\tau )dU_\tau + {\widehat{X}}_0. \end{aligned}$$

The variance may then be found by

47 $$\begin{aligned} \begin{aligned} {\mathbb {V}}[X_t|{\mathcal {G}}_t]&= {\mathbb {E}}\left[ \left( \int _0^{t}mL_{2,c}(\tau )dU_\tau + {\widehat{X}}_0\right) ^2\ \bigg |\ {\mathcal {G}}_t\right] - {\widehat{X}}_0^2\\&= \left[ \int _0^{t}mL_{2,c}(\tau )dU_\tau \right] ^2= m^2\int _0^{t}L_{2,c}^2(\tau )d\tau , \end{aligned} \end{aligned}$$

where the third equality is derived by application of Itô isometry. Similarly, for the OU process, using Eq. (18),

48 $$\begin{aligned} {\widehat{X}}_t = {\widehat{X}}_0 e^{-pt} + \int _0^t mL_{2,c} e^{-p(t-\tau )}dU_\tau , \end{aligned}$$

using a variable substitution ${\widehat{Y}}_t = {\widehat{X}}_te^{pt}$. Variance can then be found by

49 $$\begin{aligned} {\mathbb {V}}[X_t|{\mathcal {G}}_t]= & {} {\mathbb {E}}\left[ \left( {\widehat{X}}_0 e^{-pt} + \int _0^t mL_{2,c} e^{-p(t-\tau )}dU_\tau \right) ^2\ \bigg |\ {\mathcal {G}}_t\right] - \left( {\widehat{X}}_0 e^{-pt}\right) ^2\nonumber \\= & {} \left[ \int _0^{t}mL_{2,o}(\tau )e^{-p(t-\tau )}dU_\tau \right] ^2\nonumber \\= & {} m^2\int _0^{t}L_{2,o}^2(\tau )e^{-2p(t-\tau )}d\tau \end{aligned}$$

again by Itô isometry. Since both observation processes are Gaussian for all *t*, we now have all the elements necessary to describe the distributions at a given point in time.

The integral in (47) is easily solvable, but this is not the case with the integral in Eq. (49), due to the complexity of $L_{2,o}(t)$. We have therefore used simulated trials of the observation process to estimate the probability distribution variances. Specifically, if ${\mathbb {P}}$ denotes the Gaussian probability distribution of $X_t$ given previous observations at *t*, we may denote the equivalent distribution using a simulated variance as $\widehat{{\mathbb {P}}}$. By total probability,

50 $$\begin{aligned} {\mathbb {E}}\left[ V^{1-\gamma }\left( X_t\right) \right] \approx \int _{{\mathbb {R}}}V^{1-\gamma }(x)\ \widehat{{\mathbb {P}}}(X_t = x\ |\ {\mathcal {G}}_t)\ dx, \end{aligned}$$

where

51 $$\begin{aligned} \widehat{{\mathbb {P}}} \sim {\mathcal {N}}\left( {\widehat{X}}_t, \widehat{{\mathbb {V}}}\right) , \end{aligned}$$

and $\widehat{{\mathbb {V}}}$ is the sample variance of the trial data $\{{\widehat{X}}_{t, i}\}_i$ for $i = 1,\ \dots ,\ M$ given previous observations at *t*, where *M* is the number of trials such that $\underset{M\rightarrow \infty }{\lim } \widehat{{\mathbb {P}}} = {\mathbb {P}}$ by the central limit theorem.^13^

The expectation of Eq. (50) assumes that the entire project value is received at the time of investment. As noted in the introductory paragraph of Sect. 3, our model may be extended to allow for profit streams that arrive over time. The utility function in Eq. (34) would then be applied over the profit stream function, and the expectation would be taken over the integral of discounted utilities of profits. This would simplify to an integral over expectations of utilities of profits. However, the expectation is taken at time *t* for profits that arrive in the future, so we would have to derive the future distribution conditioned on a starting point at the current process estimate in order to apply total probability once again. This is not an issue, however, since for our applications, the time dependent distribution of the processes may be derived if an initial value is specified.

### D The Bellman equation

The idea behind the Bellman principle is that, in the continuation region, the project’s value, *f*, should, at any point in time, be equal to the expected discounted value of the project a small amount of time *dt* later. That is,

$$\begin{aligned} \begin{aligned} f(t,{\widehat{X}}_t)&= \mathbb {E}\Big [e^{-\rho dt}f(t+dt,{\widehat{X}}_{t+dt})\Big |{\mathcal {F}}_t\Big ]\\&=(1-\rho dt)\mathbb {E}[f(t+dt,{\widehat{X}}_{t+dt}){\mathcal {F}}_t]+o(dt), \end{aligned} \end{aligned}$$

which can be rewritten as

52 $$\begin{aligned} \rho fdt = \mathbb {E}[df]+o(dt). \end{aligned}$$

By applying Itô’s lemma the RHS of (52) can be expanded as:

53 $$\begin{aligned} \begin{aligned} \rho fdt&=\mathbb {E}[df]+o(dt)\\&=\mathbb {E}\left[ \frac{\partial f}{\partial t}dt+\frac{\partial f}{\partial {\widehat{X}}_t}d{\widehat{X}}_t+\frac{1}{2}\frac{\partial ^2f}{\partial {\widehat{X}}_t^2}(d{\widehat{X}}_t)^2\right] +o(dt)\\&=\frac{\partial f}{\partial t}dt+\mathbb {E}\left[ L_1(t){\widehat{X}}_tdt +L_2(t)dZ_t\right] \frac{\partial f}{\partial {\widehat{X}}_t}+\frac{1}{2}\mathbb {E}\left[ L_2^2(t)(dZ_t)^2\right] \frac{\partial ^2f}{\partial {\widehat{X}}_t^2}+o(dt)\\&=\frac{\partial f}{\partial t}dt+[L_1(t)+L_2(t)]{\widehat{X}}_t\frac{\partial f}{\partial {\widehat{X}}_t}dt+\frac{1}{2}m^2L_2^2(t)\frac{\partial ^2f}{\partial {\widehat{X}}_t^2}dt+o(dt). \end{aligned} \end{aligned}$$

After dividing by *dt* on both sides and by taking the limit $dt\downarrow 0$, this simplifies to a partial differential equation (PDE) that characterizes the value function in the continuation region:

54 $$\begin{aligned}&\frac{\partial f(t,{\widehat{X}}_t)}{\partial t}+[L_1(t)+L_2(t)]{\widehat{X}}_t\frac{\partial f(t,{\widehat{X}}_t)}{\partial {\widehat{X}}_t}\nonumber \\&\quad +\frac{1}{2}m^2L_2^2(t)\frac{\partial ^2f(t,{\widehat{X}}_t)}{\partial {\widehat{X}}_t^2}-\rho f(t,{\widehat{X}}_t)=0.\qquad \end{aligned}$$

### E The Longstaff-Schwartz method

Longstaff and Schwartz (2001) show that the simulated option value resulting from the algorithm outlined in Sect. 4 is bounded by the true option value from above when the number if trials *S* approach infinity, i.e.

55 $$\begin{aligned} F^*_0 \ge \lim _{S \rightarrow \infty }\frac{1}{S}\sum ^{S}_{s = 1}LSM(\omega _s), \end{aligned}$$

where $\omega _s$ indicates the *s*-th trial, and $LSM(\omega _s)$ is the discounted exercise value of $\omega _s$ when following the algorithm investment rules. Note that any set of orthonormal basis functions may be used in the regressions so long as their linear combination span the range of the continuation value. Longstaff and Schwartz (2001) use Laguerre polynomials, but point to other possibilities such as Hermite, Legendre, Chebyshev and Jacobi polynomials. They then proceed to show that the option value resulting from following the algorithm converges to the true option value when the number of basis functions increases. Hence, for a high enough number of trials, the simulated option value will approach the true option value from below when the number of basis functions increases. This result is useful in that it allows the user to iteratively increase the number of basis functions until the estimated option value $f_0$ increases from the previous estimation by an amount $\bar{\epsilon }$ that is deemed acceptably small.

Following Longstaff and Schwartz (2001), we have chosen the set of Laguerre polynomials as basis functions. Using similar notation, we denote the *n*th order polynomial as ${\mathcal {L}}_n({\widehat{X}}_j)$, expressed in closed form by

56 $$\begin{aligned} {\mathcal {L}}_n({\widehat{X}}_j) = \sum _{k = 0}^{n}\left( {\begin{array}{c}n\\ k\end{array}}\right) \frac{(-1)^k}{k!}{\widehat{X}}_j^k. \end{aligned}$$

The regression equation may then be written as follows, with ${\widehat{X}}_j$ as the regressor and $Y(\omega _i, j\Delta t)$ the regressand, at $j = \{1,\ \dots ,\ N - 1\}$,

57 $$\begin{aligned} Y(\omega _i, j\Delta t) = \sum _{n=0}^{B} a_{n,j} {\mathcal {L}}_n({\widehat{X}}_j), \end{aligned}$$

where *B* denotes the number of basis functions and $\{\varvec{a}_{j}\}$ the corresponding coefficients.

The exercise boundary is found by solving for the estimation process values that equate the regression equation—or, the conditional expectation of the continuation value—with the exercise value, at each discrete time point $j = \{1,\ \dots ,\ N - 1\}$, such that

58 $$\begin{aligned} {\widehat{X}}^*_j= \inf \left\{ {\widehat{X}}_j\in {\mathbb {R}}:\sum _{n=0}^{B} {\hat{a}}_{n,j} {\mathcal {L}}_n({\widehat{X}}_j) = 0 \right\} , \end{aligned}$$

where $\{\hat{\varvec{a}}_{j}\}$ are the estimated coefficients resulting from the regression. The free boundary at $j = N$ is set equal to the indifference value of exercising the option. Since the regression functions consist of weighted polynomials of order potentially higher than 4, and the Abel-Ruffini theorem states that such polynomials do not have an algebraic solution when equated to zero, we find the boundary points by a numerical solution procedure. After the boundary points have been identified, we apply a polynomial smoothing technique that identifies the least squares coefficients of a polynomial of user-defined order $\eta$, with fixed endpoints.^14^

### F Proof of Proposition 1

The proof strategy is similar to the proof of Olsen and Stensland (1992, Proposition 3). Suppose that ${\tilde{a}}^2=a^2+\varepsilon$, for some $\varepsilon >0$. Let ${\mathcal {A}}$ and ${\tilde{{\mathcal {A}}}}$ denote the characteristic operators under *a* and ${\tilde{a}}$, respectively. Since ${\tilde{F}}^*$ is optimal we know that

$$\begin{aligned} {\tilde{{\mathcal {A}}}}{\tilde{F}}^*\le \rho {\tilde{F}}^*. \end{aligned}$$

Let ${\tilde{L}}_i$, $i=1,2$ denote the $L_i$ function under ${\tilde{a}}$. It holds that $L_2=-L_1$ in this case, whence

$$\begin{aligned} {\mathcal {A}}{\tilde{F}}^*(t,x) =&\, {\tilde{{\mathcal {A}}}}{\tilde{F}}^*(t,x)+({\mathcal {A}}-{\tilde{{\mathcal {A}}}}){\tilde{F}}^*(t,x)\\ \le&\rho {\tilde{F}}^*(t,x)+({\mathcal {A}}-{\tilde{{\mathcal {A}}}}){\tilde{F}}^*(t,x)\\ =&\,\rho {\tilde{F}}^*(t,x)+\frac{1}{2}m^2[L^2_2(t)-{\tilde{L}}^2_2(t)]\frac{\partial ^2{\tilde{F}}^*(t,x)}{\partial x^2}\\&+[(L_1(t)-{\tilde{L}}_1(t))+(L_2(t)-{\tilde{L}}_2(t))]x\frac{\partial {\tilde{F}}^*(t,x)}{\partial x}. \end{aligned}$$

From the general theory (see, e.g., Olsen and Stensland 1992) we know that the value function is non-decreasing and convex in *x*. It has already been established that $L_2$ is increasing in *a*. A similar calculation shows that $L_1$ is also increasing in *a*. Therefore,

$$\begin{aligned} {\mathcal {A}}{\tilde{F}}^*\le \rho {\tilde{F}}^*, \end{aligned}$$

so that ${\tilde{F}}^*$ is superharmonic under *a*. It also dominates $U\circ F$. However, it is not the *least* superharmonic function that dominates $U\circ F$, because that is $F^*$. Hence, ${\tilde{F}}^*\ge F^*$. $\blacksquare$

### G Relative error against number of basis functions

Figure 7 shows the relative error of the option values plotted against the number of basis functions used in simulations of the base case. Note that the plots use different horizontal axis ranges.

### H Further robustness checks

In this section we perform some further numerical experiments to illustrate the robustness of the numerical scheme. In Fig. 8 we show the simulated investment time distributions for the constant and OU processes for different values of the scaling parameter of the initial estimate, ${\hat{X}}_0-\mu$, in the risk-neutral case. The same distributions are plotted for the risk-averse case in Fig. 9. In Figs. 10 and 11 we plot the investment time distributions for the constant and OU processes in the risk-neutral and risk-averse cases, respectively, for different values of the standard deviation of the initial estimate, *a*. In all these figures it is clear that the distribution of investment times is skewed. This is consistent with the analytical result that the first-hitting time distribution of a standard Brownian motion is skewed (see, e.g., Øksendal 2013). Since the randomness in our models are driven by a standard Brownian motion, we would expect this skewness to carry over. We also note that, qualitatively, the distributions all look similar, which we take as evidence of the method’s robustness.

## Footnotes

1 Our method in this section is largely based on the procedure presented in Øksendal (2013), Chapter 6.

2 All derivations are given in Appendix 1.

3 Derivations are given in Appendix 2.

4 Further details are given in Appendix 3.

5 An intuitive derivation of the Bellman equation can be found in Appendix 4.

6 Further details are provided in Appendix 5.

7 For comparability, we have used the same random number generator seed in all sections, specifically, rng(1108) in MATLAB v. R2020a.

8 We have selected a polynomial order of $\eta = 16$ for the smoothing function in the risk neutral case. The discrete points will be included in the initial free boundary plot to illustrate the fit, but will be omitted for convenience in later sections.

9 There are some numerical issues close to the option’s maturity, which are due to a discontinuity of the exercise boundary at time *T*. Intuitively, this happens because at time $T-$ there is still a positive value of learning that is not converging to zero. This is why we report results up to time 4.5 only.

10 Note that the value function, $\Psi (\cdot )$, under risk aversion now has a direct time dependence, as opposed to the risk neutral case, due to the time dependence of the variance in the probability distribution as detailed in Appendix 3.

11 The free boundary in the risk averse case shows some fluctuations for $t \gtrsim 1$.We have applied a polynomial order of $\eta = 10$ for the smoothing function under risk aversion. This is can be a result of inaccuracies arising from the approximation of the exercise value function.

12 This can be seen, for example, in a simple model of deterministic growth, where $dX_t=\alpha X_tdt$ and observations $dZ_t=X_tdt+\sigma dV_t$, with $\alpha ,\sigma >0$.

## References

[CR1] Barker AL, Brown DE, Martin WN (1995) Bayesian estimation and the Kalman filter. Comput. Math. Appl. 30:55–77

[CR2] Bellalah M (2001) Irreversibility, sunk costs and investment under incomplete information. R&D Manag 31(1):115–126

[CR3] Bergemann D, Välimäki J (2008) Bandit problems. In: Durlauf SN, Blume LE (eds) The new palgrave dictionary of economics, vol 1, 2nd edn. Macmillan Press, New York, pp 336–340

[CR4] Blanke D, Bosq D (2012) Bayesian prediction for stochastic processes: theory and applications

[CR5] Dalby PAO, Gillerhaugen GR, Hagspiel V, Leth-Olsen T, Thijssen JJJ (2018) Green investment under policy uncertainty and Bayesian learning. Energy 161(1):1262–1281

[CR6] Dixit AK, Pindyck RS (1994) Investment under uncertainty. Princeton University Press, Princeton, New Jersey

[CR7] Dumas B (1991) Super contact and related optimality conditions. J Econ Dyn Control 15:675–685

[CR8] Ekström E, Lindberg C, Tysk J (2011) Optimal liquidation of a pairs trade. In: Di Nunno G, Øksendal B (eds) Advanced mathematical methods for finance. Springer, Heidelberg, pp 247–255

[CR9] Financial Times (2021) The race to scale up green hydrogen. Accessed 30 Mar 2021

[CR10] Gray A, Greenhalgh D, Hu L, Mao X, Pan J (2011) A stochastic differential equation SIS epidemic model. SIAM J Appl Math 71(3):876–902

[CR11] Grewal MS (2011) Kalman filtering. Springer, Berlin, Heidelberg, pp 705–708

[CR12] Hagspiel V, Nagy RLG, Sund S, Thijssen JJJ (2019) Investment under uncertainty with costly Bayesian learning: the optimal choice of learning rate. Working paper

[CR13] Harrison JM, Sunar N (2015) Investment timing with incomplete information and multiple means of learning. Oper Res 63(2):442–457

[CR14] Henderson V, Hobson DG (2002) Real options with constant relative risk aversion. J Econ Dyn Control 27:329–355

[CR15] Herath HSB, Herath TC (2008) Investments in information security: A real options perspective with Bayesian postaudit. J Manag Inf Syst 25(3):337–375

[CR16] Hugonnier J, Morellec E (2007) Real options and risk aversion. Swiss Finance Institute Research Paper

[CR17] Hull JC (2015) Options, futures and other derivatives, 9th edn. Pearson, Boston, Massachusetts

[CR18] Jorion P, Sweeney RJ (1996) Mean reversion in real exchange rates: Evidence and implications for forecasting. J Int Money Finance 15(4):535–550

[CR19] Keller R, Rady S (1999) Optimal experimentation in a changing environment. Rev Econ Stud 66(3):475–507

[CR20] Kolm PN, Ritter G (2019) Dynamic replication and hedging: A reinforcement learning approach. J Financ Data Sci 1(1):159–171

[CR21] Krylov N (1980) Controlled diffusion processes. Springer Verlag, Heidelberg

[CR22] Kwon HD (2014) Prevention of catastrophic failures with weak forewarning signals. Probab Eng Inf Sci 28(1):121–144

[CR23] Kwon HD, Lippman SA (2011) Acquisition of project-specific assets with Bayesian updating. Oper Res 59(5):1119–1130

[CR24] Kwon HD, Xu W, Agrawal A, Muthulingam S (2016) Impact of Bayesian learning and externalities on strategic investment. Manag Sci 62(2):550–570

[CR25] Leland HE (1985) Option pricing and replication with transactions costs. J Financ 40(5):1283–1301

[CR26] Leung T, Li X (2015) Optimal mean reversion trading with transaction costs and stop-loss exit. Int J Theor Appl Finance 18(3):1550020

[CR27] Longstaff FA, Schwartz ES (2001) Valuing American options by simulation: a simple least-squares approach. Rev Financ Stud 14(1):113–147

[CR28] Lucia JJ, Schwartz ES (2002) Electricity prices and power derivatives: evidence from the Nordic power exchange. Rev Deriv Res 5:5–50

[CR29] Martzoukos S, Trigeorgis L (2001) Resolving a real options paradox with incomplete information: after all, why learn? Working paper

[CR30] McDonald R, Siegel D (1985) Investment and the valuation of firms when there is an option to shut down. Int Econ Rev 26:331–349

[CR31] McDonald R, Siegel D (1986) The value of waiting to invest. Q J Econ 101:707–728

[CR32] Mjaavatten A (2020) polyfix (v. 1.3.1.2). URL: https://www.mathworks.com/matlabcentral/fileexchange/54207-polyfix-x-y-n-xfix-yfix-xder-dydx. Accessed 27 June 2020

[CR33] Moscarini G, Smith L (2001) The optimal level of experimentation. Econometrica 69(6):1629–1644

[CR34] Näsäkkälä E, Fleten S-E (2005) Flexibility and technology choice in gas fired power plant investments. Rev Financ Econ 14(3):371–393

[CR35] Øksendal B (2013) Stochastic differential equations: an introduction with applications, 6th edn. Springer, New York

[CR36] Olsen TE, Stensland G (1992) On optimal timing of investment when cost components are additive and follow geometric diffusions. J Econ Dyn Control 16:39–51

[CR37] O’Driscoll M, Dos Santos GR, Wang L, Cummings DA, Azman AS, Paireau J, Fontanet A, Cauchemez S, Salje H (2021) Age-specific mortality and immunity patterns of sars-cov-2. Nature 590(7844):140–145 33137809 10.1038/s41586-020-2918-0

[CR38] Pertile P, Forster M, Torre DL (2014) Optimal Bayesian sequential sampling rules for the economic evaluation of health technologies. J R Stat Soc 177(2):419–438

[CR39] Peskir G, Shiryaev A (2006) Optimal stopping and free-boundary problems. Birkäuser Verlag, Basel

[CR40] Peura S, Keppo J (2005) Optimal bank capital with costly recapitalization. J Bus 79:2163–2201

[CR41] Ryan R, Lippman SA (2003) Optimal exit from a project with noisy returns. Probab Eng Inf Sci 17(4):435–458

[CR42] Schwartz ES (1997) The stochastic behavior of commodity prices: Implications for valuation and hedging. J Financ 52(3):923–973

[CR43] Singh R, Ghosh D, Adhikari R (2018) Fast Bayesian inference of the multivariate Ornstein-Uhlenbeck process. Phys Rev E 98:1–9 10.1103/PhysRevE.98.012136 30110802

[CR44] Soyer R (2018) Kalman filtering and sequential Bayesian analysis. WIREs Comput Stat 10:e1438

[CR45] Thijssen JJJ, Bregantini D (2017) Costly sequential experimentation and project valuation with an application to health technology assessment. J Econ Dyn Control 77(C):202–229

[CR46] Thijssen JJJ, Huisman KJM, Kort PM (2004) The effect of information streams on capital budgeting decisions. Eur J Oper Res 157(3):759–774

[CR47] Vasicek O (1977) An equilibrium characterization of the term structure. J Financ Econ 5(2):177–188

[CR48] Wang W, Cai Y, Ding Z, Gui Z (2018) A stochastic differential equation sis epidemic model incorporating Ornstein-Uhlenbeck process. Phys A 509:921–936

[CR49] Wong HY, Lo YW (2009) Option pricing with mean reversion and stochastic volatility. Eur J Oper Res 197(1):179–187

